# Development of the Parental Expectations and Perspectives Questionnaire in Hindi to Measure the Outcomes from Pediatric Cochlear Implantation

**DOI:** 10.1055/s-0044-1791275

**Published:** 2025-05-23

**Authors:** Mohammad Shamim Ansari, Arvinder Singh Sood, Satya Prakash Dubey

**Affiliations:** 1 Department of Audiology, Ali Yavar Jung National Institute of Speech and Hearing Disabilities, Mumbai, Maharashtra, India; 2 Department of Ear, Nose & Throat, Sri Guru Ram Das Institute of Medical Sciences and Research, Amritsar, Punjab, India

**Keywords:** hearing impairment, cochlear implant, parental expectation & perspective, questionnaire, outcomes, Hindi

## Abstract

**Introduction** The pediatric cochlear implantation (PCI) outcomes, as seen through the parental perspective questionnaire (PPQ), may provide a more comprehensive and accurate view on the functional status of cochlear implant recipients in real-life situations. However, there is no Hindi language version of the PPQ. Therefore, the purpose of the present study was to translate and culturally adapt the original PPQ into Hindi to measure the outcomes in children with CI.

**Methods** The original PPQ was translated into Hindi through a forward-backward process. The questionnaire was content- and face-validated. The harmonized Hindi questionnaire consisted of 74 items covering two domains: decision-making (26 items) and outcomes (48 items). It was piloted in 139 parents of children with CI, to determine the validity and reliability and to measure the outcome of PCI.

**Results** The PPQ-Hindi version was easy to understand for parents. They reported that the questionnaire completion time was appropriate. The instrument had a high degree of content- and face-validity, and it matched the original. The overall Cronbach α was 0.89, and the test–retest reliability coefficient was 0.92.

**Conclusions** The PPQ was successfully translated into Hindi and adapted to this specific culture and population, exhibiting a good validity and reliability to measure outcomes in PCI. Thus, the instrument has the potential to be an effective tool for parents to self-administer and to evaluate the effects of CI in their children.

## Introduction

Approximately 1.4 per 1,000 children are born with prelingual deafness in both ears worldwide. Deafness in the early years of life limits the oral language learning, educational attainment, psychosocial development, and quality of life (QoL) of children. 1 However, the negative impact of deafness in children can be reversed by fitting hearing aids or cochlear implants (CIs). The latter requires a surgical procedure to bypass the damaged sensory organ and directly stimulate the spiral ganglion cells in the cochlear modiolus, inducing sensation and perception of sound. 2 The CI has demonstrated various clinical benefits from a professional view point, such as: enhanced hearing ability, as well as improved skills, such as speech and language, communication development equal to hearing peers, age-appropriate reading level, 3 cognitive improvement, 4 and better academic performance. 5

These positive reports have heightened the rate of CI in children in recent times. 6 It has been considered as standard of care for treating hearing impairment and promoting oral language development in people with profound deafness who would not benefit from hearing aids otherwise. 2 A recent report estimates that about 1,000,000 (one million) CIs have been fitted to restore hearing worldwide, 7 a majority of them for children. 8

Regarding pediatric patients, the decision for CI is made by the patient's parents. However, considering the rate of implantation, the desired outcomes in real life situations are variable and unpredictable in this population. 9 10 Hence, the functioning of children with CI in everyday life situations, from their parents' perspectives covering various aspects of CI, have been routinely studied for the last two decades. One of such parental perspective questionnaires (PPQs) is the Parents' Views and Experiences with Pediatric Cochlear Implants Questionnaire (PVECIQ). 11

This instrument is used in many CI centers worldwide and has been described as an excellent research and clinical tool for studying the experiences and opinions of parents about several aspects of children and their family's following surgery. 11 12 The PVECIQ has been translated and adapted into more than nine languages to assess the effects of CI in children worldwide. Damen et al. 13 translated and culturally adapted this questionnaire into Dutch, Huttunen et al. 14 into Finish, Fortunato-Tavares et al. 15 and Stefanini et al. 16 into Brazilian Portuguese, Byckova et al. 17 into Lithuanian, Molla et al. 18 into Bangla, Zhao et al. 19 into Mandarin, Shah Mahmood et al. into Persian, 20 Zhumabayev et al. 21 into Kazakh and Russian, and Danielian et al. into Armenian. 22 The English version of the PVECIQ has also been adapted for different cultures and populations to study the influence of CI. Anne et al. 23 and Kumar et al. 25 adopted the PVECIQ for the United States, and Brewis et al. 24 for South Africa. These studies suggest that the questionnaire can be adapted to diverse linguistic, social, economic, and cultural populations to measure post-CI outcomes in children from a parental perspective.

Studies using this instrument have supplied additional and valuable information about various aspects of PCI in real-life settings, regarding children's functionality, efficiency of the implementation process, and need for additional interventions. This information has helped the rehabilitation team to design need-based interventions for children, provide parental counselling, and improve service. It has also helped policy makers to implement more successful CI programs. 26 27 Therefore, in recent times, parental perception is routinely evaluated through validated questionnaires in the Western literature. 16 28 29 Despite the widespread application of this instrument, there is no validated parental questionnaire available in the Indian literature, including in Hindi, to measure children's outcome from PCI, to the best of our knowledge.

Furthermore, considering the World Health Organization's 2018 estimate, India is home to the largest deaf population in the world. 30 A survey of the Social Statistics Division from the Government of India in 2016 reported that the Hindi speaking belt of Northern India has a larger population with disabilities under the age of 16 compared to rest of India's population, and deafness is the second highest disability found in the country. 31 Thus, it is expected that CI will be performed frequently than before in both the public and private sectors in India. However, in comparison to the growing rate of CI in children, the authenticated or systematic published results in this area are scarce. This may be due the lack of a valid and reliable PPQ in Hindi.

There have been sporadic studies on applications of parental perspectives, experiences, and expectations from PCI in India. Dev et al. conducted the study to understand the perspectives of parents of pediatric CI users on re/habilitation services using a non-standardized questionnaire comprising 46 items. 32 Khan & Rajguru studied parents' preimplant expectations and the postimplant experiences of their children's CI outcomes using a Hindi questionnaire based on the International Classification of Functioning, Disability, and Health for Children and Youth (ICF-CY), specially the items related to domains pertaining to listening, communication, learning and applying knowledge, interpersonal interactions and relationships, and environmental factors. 33 Sud et al. interviewed parents to know the child's needs and experiences with CI using the closed-format Strengths and Difficulties Questionnaire (SDQ) in Hindi. 34

However, to the best of our knowledge, the authors did not report the validity, reliability, or feasibility of the application in the target population. Thus, to strengthen these efforts, there is a need to develop a validated and reliable PPQ in Hindi to measure the CI outcomes in children. Therefore, the purpose of the current study was to develop an independent, closed-format, PPQ in Hindi.

## Methods

The study received ethics approval from the Ethics Review Committee of Sri Guru Ram Das University of Health Sciences. A simple prospective and retrospective (mixed) survey research design was chosen. The purposive sampling technique was used to select the participants. We invited a total of 139 Hindi-speaking parents of children with CI, between 3 and 9-years old. All children underwent implantation before 6 years old, with continuous CI use for at least 2 years. Furthermore, they were all enrolled in the post-CI rehabilitation program. The model and brand of CI device used was not considered for analysis.

Participation was voluntary, and written consent forms were obtained from all participants. The sample were selected from CI centers in Mumbai, Amritsar, Bhopal, Meerut, Lucknow, and others, where parents visited for routine follow-up consultations and rehabilitation programs for their children.

**Fig. 1:**
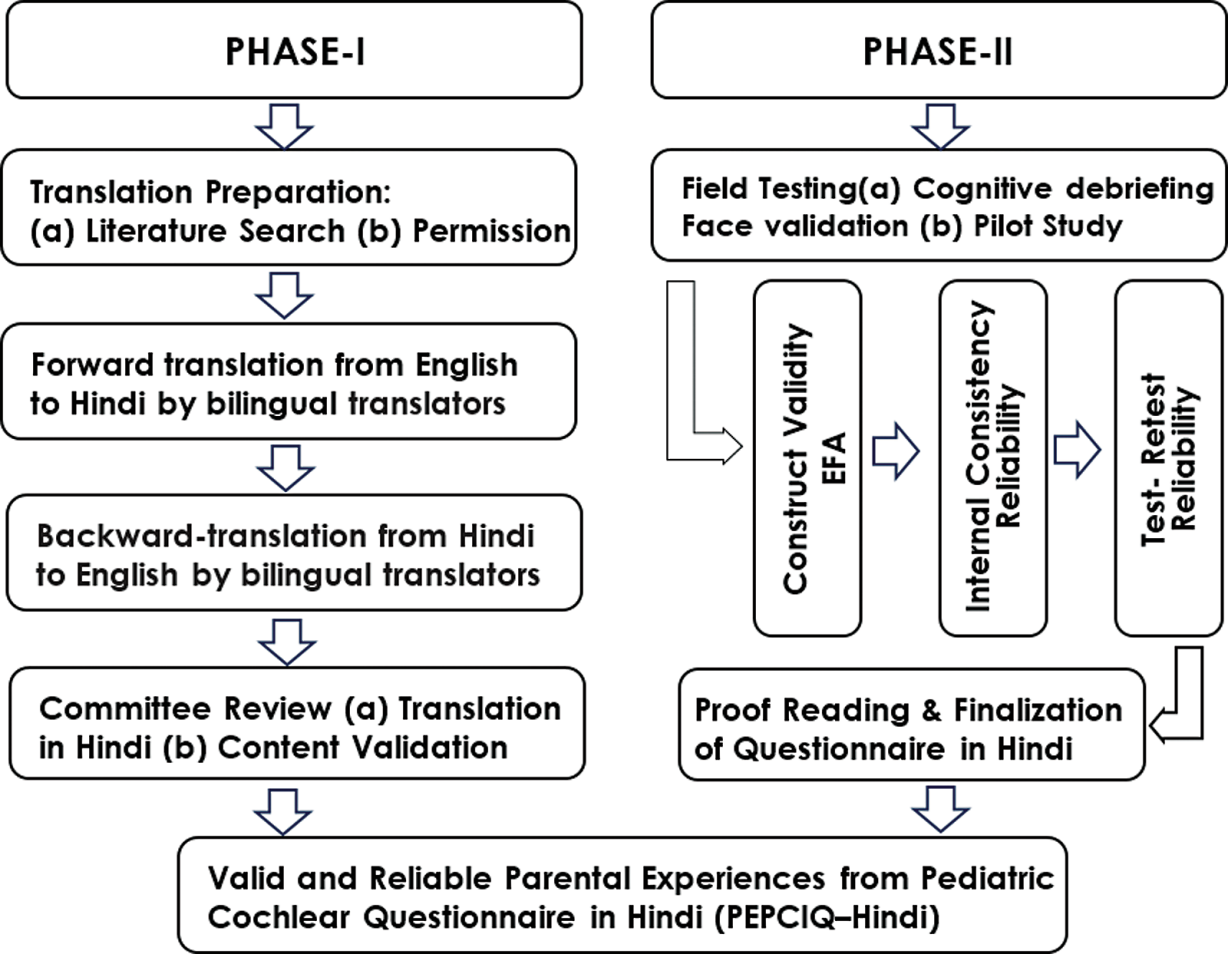
Showing sequential steps divided into two phases involved in the translation process.

The translation and cultural adaptation of the PPQ in Hindi was conducted according to the norms of the backward-forward system, 35 in six sequential steps divided into two phases ( Fig. 1 ):

- Phase I: (1) Translation preparation. (2) Forward translation. (3) Back translation. (4) Committee review & content validation.
- Phase II: (5) Field testing – face-validation & pilot testing, construct validation & reliability testing. And (6) Reviewing & finalizing the translation.

### Phase I

#### Translation Preparation

The literature search indicated that the PVECIQ is not available in Hindi and the original English questionnaire is a copyrighted material, owned by Elsevier Publisher. Thus, the permission to translate this questionnaire into Hindi had to be obtained, being granted under agreement License Number 4111901195442.

#### Forward Translation: from Source into Target Language

For forward translation, two bilingual translators whose mother tongue was Hindi, with live-in experience of the target culture and country, were selected to produce independent translations of the original instrument into Hindi, the target language. One of them was a professional translator with certified linguistic competency but unaware of the motive behind the translation of the instrument, and the second was an audiologist, experienced with CI, relevant rehabilitation terminologies, and who was aware of the translation's purpose. This mix in set was designed to maintain a linguistic and conceptual equivalence between the original and target languages. This balance in skill set was also intended to reduce the individual biases and promoting a translation that can fulfill its purpose. The translators were requested to use plain, clear, and concise language, which suits the culture of the Hindi-speaking population.

The translated versions were reconciled into an optimal Hindi version. For this purpose, a third translator, an audiologist with 5 years of practicing experience with CI and translation of a hearing-related QoL questionnaire was selected. He was required to maintain the conceptual contents, items, and semantic equivalences, and to use everyday, nontechnical language in the reconciled translation. The two forward translations were compared and assessed in terms of quality simplicity and clarity of words, minimizing the amount of technical and specialized words for the general public, and maintaining the conceptual content of the original version. He was also requested to assess the differences in cultures and linguistics that may cause difficulties or ambiguity in the Hindi version. Both translations were reviewed item by item, and the best one was selected or an alternative suggested when appropriate.

In this manner, the third translator generated a reconciled version of the forward translation in Hindi.

#### Backward Translation: from Target to Source Language

In this step, the reconciled version in the target language was back translated on a unit-by-unit basis, for all parts of the instrument in its original language, that is, English. A bilingual speaker with appropriate linguistic expertise, experience with the disease/disorder in interest, and with live-in experience of the Indian culture for 10 years was appointed as back translator. He was given only the reconciled translation in Hindi and blinded to the existence of the original questionnaire to reduce the potential of ambiguous translations.

#### Committee Review

The review committee or expert panel should include the original developer of the instrument, as well as healthcare providers with experience in instrument development and translation. 36 37 Since the authors of the original instrument could not be reached and may not be proficient in translation, they were not invited to the committee.

The review committee consisted of a linguistic expert with Hindi language expertise; a healthcare professional with in-depth knowledge of the field, content area, and research methodology & translation process; all the forward and backward translators; and the lead researcher as committee leader. The committee discussed the results of all translations and, when necessary, modified sentences to better suit the Indian cultural values, and offered appropriate suggestions to achieve equivalence between the original and translated versions. The leader incorporated all necessary suggestions of the committee at this step of translation. By doing so, the instrument was developed in the Hindi language.

##### Content Validation

The Hindi questionnaire was subjected to content validation, which was accomplished by 6 experts with 10 years of experience minimum in the area. The expert team consisted of two speech language pathologists, two audiologists, and two CI surgeons. The experts reviewed the original and translated items for conceptual equivalence. This process also identified potentially problematic survey items and translation errors. The validators were asked to review and rate the individual statements/items on a 4-point scale: 1 - very suitable; 2 - suitable; 3 - suitable but needs modification; and 4 - not suitable. The statements/items rated 1 or 2 by experts were selected and those marked as 3 or 4 were removed from the questionnaire. The experts also determined whether the questionnaire's contents were relevant to the local context and whether the items were sufficient to represent each domain. In this manner, the instrument was synthesized in Hindi.

### Phase II

#### Field Testing

Field testing was the last stage of the translation and cultural adaptation process before producing the questionnaire's final version. The questionnaire was field tested in the target population through a mixed-method design with conceptual understandings.

First, a qualitative approach was used, in this case a cognitive debriefing, and the feasibility of the questionnaire's application was determined through face-to-face semi-structured focus group interview. This was followed by a pilot study as the chosen quantitative approach, in hopes of better understanding the instrument, with an assumption that the qualitative approach would provide richer personal answers and varied written or oral comments. Likewise, a quantitative approach can determine the number of missing answers (nonresponse), and a larger sample could add to the data.

Furthermore, both approaches could give various perspectives of the target population and provide support in wording items clearly. Both methods were implemented to improve the fidelity of the questionnaire's final translated version.

##### Cognitive Debriefing-Face Validity

To test the feasibility of questionnaire application in the target population, the qualitative and quantitative face-validity was determined. At first, 15 parents of children with hearing impairment who were naive to the study were recruited to self-administer the questionnaire and respond on clarity, readability of the instructions, language and wording, content, and the general structure. Parents were also asked to offer suggestions, if willing to do so.

The quantitative face-validity was determined by calculating the items' impact score (frequency x importance) to explain the proportion of participants who distinguished between important or quite important. Items were regarded as appropriate if they had an impact score equal to or > 1.5. Parents' qualitative opinions and quantitative responses were recorded, assessed, and incorporated into the questionnaire after consultation with the experts.

##### Pilot Study

The pilot study was conducted for two purposes. First, to explore how a larger population interacts with questionnaire and how much time they take to complete the instrument. Secondly, to determine its psychometric properties before application in clinical research.

A total of 139 parents whose children had congenital or acquired hearing loss before 3-years of age and using the CI continuously at least for 2 years were recruited. The sample was adequately represented across the target population in terms of the severity of hearing loss, age, gender, education, regional dialect, socioeconomic status, and other relevant cultural factors. The questionnaire was presented to individual parents, to be filled out separately in a single session. The participants were allowed to consult each other on how to respond. However, it was requested that they arrive on a consensus to rate the item in question. They were also allowed to consult the investigator in case of confusion.

The psychometric properties of the target questionnaire were explored, including construct validity (the extent to which the questionnaire measures what it is supposed to measure) and reliability (the degree of measurement precision).

Explorative factor analysis (EFA) was performed using a principal axis analysis with a varimax rotation to validate and determine the underlying construct of the translated questionnaire. It was used to test whether the translated items agreed with the original questionnaire's constructs. The study examined two types of reliability measures, with them being internal consistency and test–retest reliability. The Cronbach alpha coefficient was explored through average correlation between the items to determine internal consistency of the questionnaire. To establish the test–retest reliability, the target questionnaire was readministered after an interval of 2 weeks to 25 parents. The correlation between the total scores of both results was determined through the ICC. The minimum acceptable value for the Cronbach alpha coefficient for internal consistency was set at equal or above 0.7. 38

#### Reviewing and Finalizing the Translation

The field-testing data, that is, cognitive debriefing, face-validity, and pilot study, were used to ensure that the questions and rating scales were culturally appropriate and correctly interpreted by the parents before proofreading. The questionnaire was revised and corrected for spelling and grammar mistakes. Then, it was proofread and certified as the final version, named as parental experiences from pediatric cochlear implant questionnaire (PEPCIQ) Hindi.

## Results

The translation and cultural adaptation of the PPQ in Hindi was successfully performed according to the norms of the backward-forward translation system, as “good practice guidelines for translating and adapting hearing-related questionnaires for different languages and cultures”. 35 The translation & cultural adaptation was accomplished in six sequential steps divided into two phases ( Fig. 1 ):

- Phase I: (1) Translation preparation. (2) Forward translation. (3) Backward translation. (4) Committee review & content validation.
- Phase II: (5) Field testing – face validation; pilot testing – construct validation; & reliability testing. And (6) reviewing & finalizing the translation.

### Phase I

The translation and cultural adaptation of the PEPCIQ was faithfully accomplished in the Hindi language and population. The qualitative content validity of the Hindi translated questionnaire was found to be suitable for the construct of interest. The quantitative content validity, analyzed by the content validity ratio (CVR), and index (CVI), based on the opinions of 6 experts, were found to be 0.89 and 0.99, respectively. This value is satisfactory and acceptable according to the Lawshe critical table. Thus, the overall results indicated that the Hindi questionnaire had acceptable content validity for application.

### Phase II

#### Field Testing

The qualitative face-validity findings showed that most items were validated by the participants. Out of the 74 translated items, 72 (97.29%) were easy to read and understand, as well as relevant to the interest area. However, 2 (0.2%) items were not easily comprehensible to the parents. They were both modified, without affecting the content and intent of the original. As an example, the word vidutiyya (electrical) was difficult to understand for some participants. In this case, the original English word was retained between parentheses to improve sentence clarity and comprehension. The quantitative face validity analysis revealed that the impact score for all the items were found to be ≥ 1.5. Thus, all translated items are appropriate for application.

Thus, the adapted content and face-validated PPQ in Hindi contained the decision-making (26 statements) and outcomes of implantation (48 statements) domains. The decision-making for CI domain is subdivided into 10 items, while 16 items assess the implantation process. The outcomes of implantation domain was divided into 8 subdomains: communication (5 items), general functioning (5), wellbeing (5), self-reliance (4), social relations (8), education (8), effects of implantation (6), and support for the child (7). There are 46 positive and 28 negative statements. Parents can share their opinions about every statement on a Likert scale ranging from 1 to 5, with 1 being strongly disagree, 2 - disagree, 3 - neither agree nor disagree (neutral), 4 - agree, and 5 - strongly agree. This translated PPQ in Hindi will be referred to as the PEPCIQ hereafter, and it is presented in Appendix I .

The pilot study of the Hindi-PEPCIQ was performed in a larger sample of 139 parents of children with CI to test the feasibility of application and completion duration. Parents were provided the printed questionnaire and were asked to express their opinion on the Likert scale regarding the outcome of CI in their children.

##### Demographics of Parents & Families

The total of 139 parents (mean age 35.27 ± 7.28 years), consisting of 99 (71.22%) mothers and 40 (28.77%) fathers, rated the outcomes of CI in their children on the Hindi-PEPCIQ version independently. The parents, according to the socioeconomic strata, were found to belong in the middle-income category (20%), followed by the low-to-middle (26.66%) and low-income (42.22%) categories. The schooling level of the respondents was high school (54.44%), higher secondary (23.33%), and higher education (20.22%). The parents provided the rating for 97 boys and 42 girls with a mean ± standard deviation (SD) age of 5.4 ± 4.3), in the range of 3 to 9-years-old. All the children were using monaural CI in the right ear. The mean chronological age was 6.77 ± 1.65 years, the mean age of implantation was 4.98 ± 1.28, and of CI use was 2.41 ± 2. 32.. The demographic data of the children with CI, provided by their parents for this study, are shown in Table 1 .

**Table 1:** The demographics of children with CI for whom parents rated the outcomes on the PEPCIQ-Hindi version

Demographics (years)	Mean	SD	Range
Age at the time of the study	6.77	1.65	3.5 − 9.7
Age at the time of CI	4.98	2.25	1.1 − 11.1
CI experience duration	2.41	2.32	2.1− 4.6

**Abbreviations:** CI, cochlear implant, SD, standard deviation.

Among the children, 93% attended speech & language therapy sessions at least twice a week, while 7% of them received speech & language therapy sessions every 15 days due to nonavailability of skilled professionals in their city. From the total, 90% were enrolled in regular school. As for scholarity, 64% of the children were in preschool, 33% in primary school, and the remaining 3% were not yet old enough to join preschool. The mean completion duration of the questionnaire by the respondent was found to be 65 ± 10 minutes, with the minimum time being 55 and the maximum 78 minutes. The mean CI assisted pure-tone average threshold for frequencies 500 Hz, as well as 1, 2, and 4 kHz in open field audiometry was 27 dB (min: 20, max: 50 dB).

##### The PEPCIQ – CI Decision-Making Subscales

The rating of the CI process subscale revealed that 79% of parents were stressed while waiting for the results of the preimplant evaluation. The decision to proceed with CI was the most challenging for 71% of parents. Furthermore, 47% of parents chose CI so that their child “would have a chance to become part of the hearing world.” Nonetheless, almost all parents desired to receive all possible information and advice before the procedure. Probably for this reason, about 78% of parents suggested that prospective families should meet and consult families with a child who is a CI user. Also, 74% of parents reported that the implantation process was not as disturbing as they had expected; almost all parents feared it might break down and were concerned about uninterrupted functioning, and wished that the Implant Centre staff could visit the child at home or at school regularly to check its functioning, or at least when called.

##### The PEPCIQ – CI Outcome Subscales

The analysis of grant showed the percentage of parental rating of 3 or more on the Likert scale was of 74.8 ± 13.5% to CI outcomes – 8 subscales of the PEPCIQ. The mean parental rating scores for average or better change in their children's condition after CI on each of the 8 subscales is depicted in Table 2 .

**Table 2:** Parental perspective mean score, standard deviation, and range in eight domains of the PEPCIQ Hindi version

	Communication	General function	Self- reliance	Wellbeing & happiness	Social relations	Education	CI effects	Support
**Mean**	3.7	3.3	3.0	3.3	3.0	3.2	2.9	2.7
**SD**	0.34	0.51	0.87	0.87	0.69	0.91	0.60	0.45
**Range**	3 − 5	3 − 4	2 − 4	3 − 5	2 − 4	3 − 4	2 − 4	2 − 4

**Abbreviations:** CI, cochlear implant, SD, standard deviation.

**Fig. 2:**
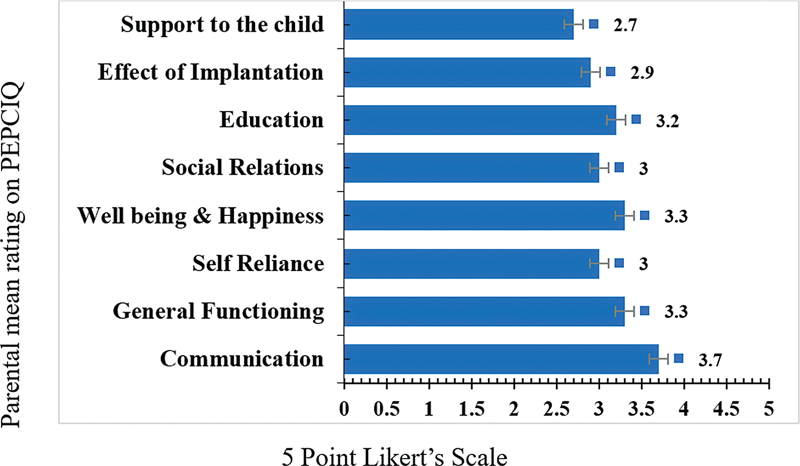
Mean score with error bar rating on 5-point Likert scale of subscales of PEPCIQ.

The communication, social relations, and self-reliance subscales showed the highest mean score percentage, whereas the effect of implantation and child support had the lowest scores. The mean score with an error bar of parents' rating of PEPCIQ subscales is represented in Fig. 2 .

The total mean parental rating for the communication, general functioning, wellbeing & happiness, and education subscales exceeded a rating of 3 on the 5-point scale, demonstrating a perceived positive change post-CI. Parents gave the highest rating points to communication (3.7 ± 0.34), followed by the child's general functioning (3.3 ± 0.51), wellbeing & happiness (3.3 ± 0.88), and education (3.2 ± 0.91). The subscales social relations (3 ± 0.69), self-reliance (3 ± 0.87), CI effect (2.9 ± 0.71), and child support (2.7 ± 0.93) had the lowest scores.

**Fig. 3:**
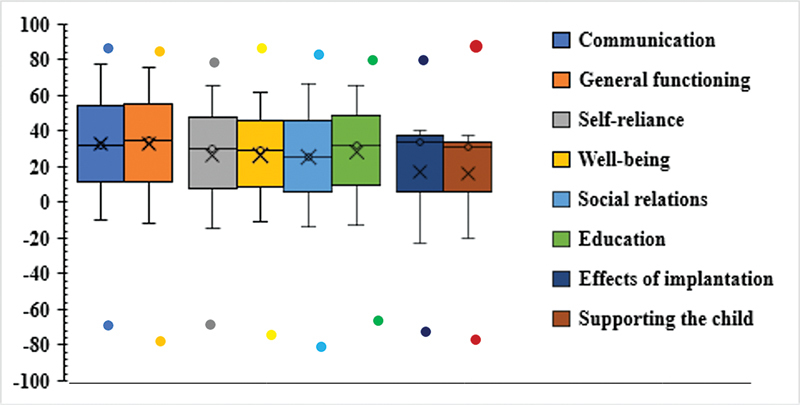
Boxplots showing the descriptive analysis of parents' rating on PEPCIQ subscales, consisting of means, median, minimum and maximum measures, and quartiles.

The response distribution on the PEPCIQ subscales exhibited by parents are depicted in Fig. 3 , showing the descriptive analysis of the means, median, minimum and maximum measures, and quartiles.

The box's bottom portion represents the first quartile, in which 25% fall below Q1, and its higher portion represents the third quartile, in which 75% fall beneath Q3. The median is depicted by the internal horizontal box, and score points above or below it with values greater than Q3 and Q1 are considered abnormal or could be outliers. The vertical lines show the observed maximum and lowest values. In the boxplot, the means of the subscales' percentages are shown as a sign of “multiplication.” The communication and self-reliance subscales indicated the highest median (45%), followed by general functionality, and wellbeing & happiness (39%). The subscales socialization, education, and CI effect obtained a median of 22%. The lowest median was obtained for the subscale support to the child (13%), meaning that parents reported the lowest score for CI supporting the child.

##### The PEPCIQ – Correlation of Different Subscales

The correlation between the subscales of the PEPCIQ was studied using the Spearman correlation test. Table 3 displays the Spearman correlation coefficient and the level of significance between the parents' and subscales' overall scores.

**Table 3:** Spearman correlation value between different subscales of the PEPCIQ

	General function	Self-reliance	Wellbeing & happiness	Social relations	Education	CI effects	Support
**Communication**	0.638*	0.604*	0.581*	0.423*	0.501**	0.317	0.520*
**General functioning**		0.542**	0.485*	0.621*	0.210	0.094	0.279
**Self-reliance**			0.167	0.563**	0.464*	0.415*	0.288
**Wellbeing & happiness**				0.232	0.155	0.008	0.566*
**Social relations**					0.432*	0.187	0.610*
**Education**						0.485*	0.233
**CI effects**							0.212

**Note:** *Statistically significant correlation when *p* < 0.05. ** Statistically significant correlation when *p* < 0.01.

Table 3 shows that communication had positive correlations with other subscales. The highest correlations were with general functioning of the child ( *r*_s_ = 0.638, *p* < 0.05), self-reliance ( *r*_s_ = 0.604, *p* < 0.05), wellbeing & happiness ( *r*_s_ = 0.581, *p* < 0.05), and education ( *r*_s_ = 0.501, *p* < 0.01). General functioning had the strongest correlations with social relations ( *r*_s_ = 0.621, *p* < 0.05), wellbeing and happiness ( *r*_s_ = 0.485, *p* < 0.05), and self-reliance ( *r*_s_ = 0.423, *p* < 0.05). Self-reliance had the strongest correlations with social relation ( *r*_s_ = 0.463, *p* < 0.01), education ( *r*_s_ = 0.464, *p* < 0.05), and CI effect ( *r*_s_ = 0.415, *p* < 0.05). Wellbeing & happiness also had a correlation with support to the child ( *r*_s_ = 0.566, *p* < 0.01).

Social relation had weak-to-moderate correlations with communication ( *r*_s_ = 0.423, *p* < 0.05), wellbeing & happiness ( *r*_s_ = 0.232, *p* < 0.05), general function ( *r*_s_ = 0.49, *p* < 0.01), effects of implantation ( *r*_s_ = 0.415, *p* < 0.05), and support of the child ( *r*_s_ = 0.233, *p* < 0.05). Education had three significant correlations with communication ( *r*_s_ = 0.501, *p* < 0.01), self-reliance ( *r*_s_ = 0.464, *p* < 0.01), and social relations ( *r*_s_ = 0.432, *p* < 0.05).

##### Construct Validity

The result of the EFA revealed that the Kaiser-Meyer-Olkin (KMO) test was moderate (0.67), and the Bartlett test of sphericity was statistically significant (x ^2^ = 781.23, df = 105, *p* < 0.05), suggesting that the data were appropriate for factor analysis. The Kaiser criteria data extraction factor with an eigenvalue of more than one showed that the PEPCIQ included three factors matching the original version, those being parental experiences towards the child's functioning in social situations, CI effect, and the required long-term support to the child. These factors were obtained through the EFA before and after rotation, as presented in Table 4 .

**Table 4:** Exploratory factor analysis of PEPCIQ for the 8 scales based on 3 components before and after rotation

PEPCIQ (domains)	Component 1		Component 2		Component 3	
	Functioning in social situations		CI effect		Long-term support	
	Before	After	Before	After	Before	After
**Communication skills**	0.76	0.69				
**General functioning**	0.71	0.63				
**Self-reliance**	0.70	0.66				
**Wellbeing & happiness**	0.66			0.57		
**Social relations**	0.67			0.68		
**Education**	0.55			0.51		
**CI effects**			0.63			0.79
**Support**					0.61	0.58

**Abbreviations:** CI, cochlear implant, PEPCIQ, parental experiences from pediatric cochlear implantation questionnaire.

The first factor, attitudes towards the implant process, had an eigenvalue of 2.1 and 54.9% of the variance. The second, functioning of children in social situations, and third, long-term support required, factors had eigenvalues of 1.8 and 1.5 and were allocated 18.9 and 18.4% of the variance, respectively. In total, the 3 extracted factors represented 58.4% of the total variance of the PEPCIQ variables.

##### Reliability of PEPCIQ

A test–retest was carried out with a 2-week interval on 35 out of 139 parents, to determine the internal and external reliability of PEPCIQ. It showed excellent reliability, with an intraclass correlation coefficient (ICC) of 0.89 (95% confidence interval [CI]: 0.88–0.89). The Cronbach alpha internal consistency was 0.92, varying between 0.91 and 0.93 for all PEPCIQ subscales. A significant correlation between test and retest scores was demonstrated through the nonparametric Wilcoxon Z score ( *p* ≤ 0.05), as shown in Table 5 .

**Table 5:** Comparison between the means and correlation of the results from the test–retest for participants

	Test (n = 139)	Retest (n = 35)	Wilcoxon test*	Spearman correlation (r)
**PEPCIQ**	78.2 ± 17.9	76.9 ± 16	0.79	0.89**

**Abbreviations:** CI, cochlear implant, PEPCIQ, parental experiences from pediatric cochlear implant questionnaire. **Notes:** *Z-scores at *p* = 0.04. **Statistically significant at * *p* < 0.05.

## Discussion

This was the first study conducted to translate and culturally adapt the original PPQ-PVECIQ into the Hindi language and Indian culture. The translation and cultural adaptation were successfully accomplished as per the norms of forward–backward translation method, commonly used for translation and adapting the health-related QoL questionnaire.

The final form of the PEPCIQ exhibited average CVR and CVI values of 0.87 and 0.98, respectively. According to the Lawshe Critical Value Table, the acceptable CVR and CVI for the 6 experts are 0.83 and 0.90, respectively. The average item impact score was found to be 3.71, which is greater than the critical acceptable value of 1.5, indicating that the translated questionnaire has an acceptable degree of face validation. Thus, the study found a high level of content- and face-validity for the Hindi-PEPCIQ to measure the construct of interest. Furthermore, these finding agree with the content validity values of PVECIQ reported by Nunes et al. 11 and the Dutch version of the questionnaire, translated and adapted by Damen at el.. 13

An inquiry into the feasibility of the questionnaire's administration and completion time revealed that instructions/items/response scale and length were considered appropriate by the participants. Parents perceived the highest improvement in communication and the lowest in support to the child. The highest rated subscale for the most positive CI effect was communication, followed by general functioning, wellbeing & happiness, education, social relations, self-reliance, and CI effect. The findings of the current study are in concurrence to many previous studies which have reported significant positive improvement in the communication and other QoL domains following CI in children. 14 16 17 22 25 39 40

The statistical analysis using the Spearman correlation coefficient revealed highest correlations in the communication and general functioning subscales. Communication was correlated with other subscales, including general functioning, wellbeing & happiness, self-reliance, social relations, CI effects, and education. These results indicated that better communication correlates with increased self-reliance and/or independence in children, as well as improved interactions between the child and friends and family members (social relations). The general functioning is positively related with communication, wellbeing & happiness, social relationships, and education. Both CI effect and support to the child had the least correlation with other outcomes, probably indicating that these children need family support irrespective of the improvement in the other areas assessed on the designated questionnaire scale.

Regarding the construct validity of the translated questionnaire, the EFA indicated that three important factors, called parental experiences towards the child's functioning in social situations, CI effect, and the required long-term support items of the translated PEPCIQ, agreed with the original questionnaire constructs. The study of Damen et al. 13 reported three domain structures in their questionnaire, differing from this study in the following factors: general communicative functioning, social and educational functioning, and CI effects. However, this is not a major concern, as the difference could be attributed to demographic characteristics of the parents and children with CI. Thus, it can be inferred that our questionnaire's ability is comparable with that of the original when measuring outcomes of children with CI in this specific population.

Finally, the PEPCIQ showed excellent reliability with an intraclass correlation coefficient (ICC) of 0.89 (95% CI: 0.88–0.89). The Cronbach alpha internal consistency total score was 0.92, varying between 0.91 and 0.93, for all subscales of the PEPCIQ. The external consistency or reliability of the instrument on two consecutive trials after 2 weeks has a Wilcoxon sign z score of 0.79 at *p* = 0.04, and the Spearman correlation coefficient (r = 0.79, at *p* = 0.04) indicated a high degree of external consistency. These results are in consensus with previous studies that have examined the parental prospective questionnaire's reliability. 11 12 Thus, our findings reflect that the translated PEPCIQ has high level of reliability and reproducibility that would be useful when gathering and interpreting data, predicting results, and measuring outcomes with acceptable accuracy and consistency.

## Conclusion

The translated and culturally adapted Hindi-PEPCIQ was found to have acceptable validity & reliability to obtain the parental perspectives to various aspect of PCI. The questionnaire can effectively evaluate parents' expectations and perspectives of their children with CIs in three broad domains, the implantation process, functioning in social situations, and long-term support. Hence, it can be expected that the results obtained from the PEPCIQ would be valuable for parents, clinicians, and researchers in the measurement of outcomes from PCI. Therefore, we recommend the use of this questionnaire to evaluate the consequences of implantation in children.
