# Supplementary material for: Development of the Parental Expectations and Perspectives Questionnaire in Hindi to Measure the Outcomes from Pediatric Cochlear Implantation
Source: Int Arch Otorhinolaryngol. 2025 May 23;29(2):1–10. doi: 10.1055/s-0044-1791275 (PMC12101876; doi:10.1055/s-0044-1791275)
Supplement: Supplementary file 1 — Supplementary Material [file 10-1055-s-0044-1791275-s2023111669or.pdf]

APPENDIX-I

Parental Expectations from Paediatric Cochlear Implantation Questionnaire (PEPCIQ) in Hindi

दिशानिर्देश:

कृपया दिशानिर्देशों को ध्यानपूर्वक पढ़िए।

1. नीचे लिखे वाक्य, कॉकेलेअर रोपण (इंप्लांट) के उपरान्त आपके बच्चे के विकास एवं घरेलू (पारिवारिक) मामलों (गतिविधियों) में आए परिवर्तन से सम्बन्धित है।

2. आपके बच्चे के व्यवहार में हुए बदलाव (विकास) एवं घरेलू (पारिवारिक) मामलों में आए परिवर्तन को 5 - पॉइंट मापनी से दर्शाया गया है। 5-पॉइंट मापनी, हर वाक्य के समांतर दी गई है। उदाहरण के लिए

संप्रेषण	द्द्ढ़तापूर्वक सहमत	सहमत	ना असहमत ना ही सहमत	असहमत	द्द्ढ़तापूर्वक असहमत
1. अब बच्चा पहले की तुलना में अधिक बातचीत करता है।	☑	☐	☐	☐	☐

3. कृपया प्रत्येक वाक्य को ध्यान से पढ़ें। और बच्चे के विकास एवं घरेलू (पारिवारिक) गतिविधियों को सही ढंग से दर्शाने वाले 5 - पॉइंट मापनी के किसी एक मापक्रम (विकल्प) का चयन करें और उस पर सही (✓) का निशान (चिन्ह) लगाएं।

4. कृपया सभी वाक्यों के समानान्तर दिए गए मापक्रम का चयन सही - सही करें। अगर किसी कारणवश आप का चयन किये हुए मापक्रम को बदलना चाहते हों तो उस पर X का निशान (चिन्ह) लगाएं और नए जवाब पर सही (✓) का निशान (चिन्ह) लगाएं। उदाहरण के लिए

**संप्रेषण**	द्द्ढ़तापूर्व सहमत	सहमत	ना असहमत ना ही सहमत	असहमत	द्द्ढ़तापूर्वक असहमत
1. अब बच्चा पहले की तुलना में अधिक बातचीत करता है।	☑	☐	☒	☐	☐

5. यदि कोई वाक्य स्पष्ट न हो। या एक से ज्यादा माप क्रम सही लगे तो सबसे उचित लगने वाले किसी एक ही माप क्रम पर सही (✓) का निशान (चिन्ह) लगा कर अपने राय (विचार), वाक्य के सामने व्यक्त करें।

6. यह प्रश्नावली आपकी परीक्षा लेने के लिए नहीं तैयार की गई है। यह प्रश्नावली आप के बच्चे में होनेवाले विकास के सम्बन्ध में आपकी राय (विचार) जानने के लिए है। अतः सभी प्रश्नों का उत्तर (जवाब)/ राय (विचार) निःसंकोच दें।

कृपया निम्न वाक्यों को ध्यानपूर्वक पढ़ें और उचित मापक्रम पर (✓) का निशान लगायें। यदि कोई वाक्य समझ न सकें तो कृपया हमसे संपर्क करें।

**COMMUNICATION**	**संप्रेषण**	द्द्ढ़तापूर्वक सहमत	सहमत	ना सहमत ना ही असहमत	असहमत	द्द्ढ़तापूर्वक असहमत
1. Communication is difficult even with people he knows well	1. परिचित लोगों के साथ भी संप्रेषण में मुश्किल होती है।	☐	☐	☐	☐	☐
2. The quality of his speech gives me cause for concern	2. इसके वाचा की गुणवता मेरी चिंता का कारण है।	☐	☐	☐	☐	☐
3. His use of spoken language has developed greatly	3. इसके बोलने की (मौखिक) भाषा का बहुत अधिक उन्नति हुई है।	☐	☐	☐	☐	☐
4. He communicates easily and effectively using his implant	4. इम्प्लांट के प्रयोग से वह संप्रेषण सरलता और सफलता से कर पा रहा है।	☐	☐	☐	☐	☐
5. Before implantation he obtained no benefit at all from his hearing aids	5. इम्प्लांट से पहले किसी भी श्रवण यंत्र से इसको सुनने में कुछ भी फायदा नहीं हुआ था।	☐	☐	☐	☐	☐

**SOCIAL RELATIONSHIP**	**सामाजिक संबंध**	द्द्ढ़तापूर्वक सहमत	सहमत	ना सहमत ना ही असहमत	असहमत	द्द्ढ़तापूर्वक असहमत
1. He was socially isolated before getting his implant	1. इम्प्लांट से पहले वह समाज से अलग-थलग था।	☐	☐	☐	☐	☐
2. Now he is talkative and engages others in conversation	2. अब वह बातें करता है तथा दूसरों को अपने बातचीत में व्यस्त रखता है।	☐	☐	☐	☐	☐
3. He is sociable within the family	3. अब वह परिवार के लोगों से काफी मिलनसार है।	☐	☐	☐	☐	☐
4. He does not make friends easily outside the family	4. वह परिवार के बाहर के लोगो को आसानी से दोस्त नहीं बनाता।	☐	☐	☐	☐	☐
5. He shares in family situations more than before implantation	5. वह पारिवारिक परस्थितिओं में, इम्प्लांट से पहले की अपेक्षा अधिक भागीदारी करता है।	☐	☐	☐	☐	☐
6. He does not have a close relationship with his grandparents	6. दादा दादी के साथ उसके नजदीकी संबंध नहीं है।	☐	☐	☐	☐	☐
7. He takes part in family relationships on an equal footing with other members	7. वह पारिवारिक मामलों में, परिवार के दूसरे सदस्यों की तरह ही, समान स्तर पर भाग लेता है।	☐	☐	☐	☐	☐
8. His relationship with brothers and sisters has improved	8. भाई और बहन के साथ उसके संबंधों में काफी सुधार हुआ है।	☐	☐	☐	☐	☐

 © 2025. The Author(s).

GENERAL FUNCTIONING	सामान्य कामकाज	दृढ़तापूर्वक सहमत	सहमत	ना सहमत ना ही असहमत	असहमत	दृढ़तापूर्वक असहमत
1. He is totally reliant on his implant all the time	1. वह हर समय अपने इम्प्लांट पर निर्भर रहता है।	☐	☐	☐	☐	☐
2. I can now let him play outside as he is aware of the sound of traffic	2. मैं उसे बाहर खेलने देता हूँ। क्योंकि वह अब यातायात की आवाज से अवगत/ जागरूक है।	☐	☐	☐	☐	☐
3. He knows when I want his attention because he can hear me call	3. वह यह जानता है कि मुझे कब उसका ध्यान चाहिए। क्योंकि अब वह मुझे सुन सकता है।	☐	☐	☐	☐	☐
4. He is still unable to cope in new situations	4. वह अभी भी नई परिस्थितियों का सामना करने में असमर्थ है।	☐	☐	☐	☐	☐
5. He can now amuse himself listening to music or watching TV or playing games	5. वह अब गाने सुन कर, टेलीविज़न देख कर तथा खेलकर अपना मन बहलाने लगा है।	☐	☐	☐	☐	☐

SELF-RELIANCE	आत्मनिर्भरता	दृढ़तापूर्वक सहमत	सहमत	ना सहमत ना ही असहमत	असहमत	दृढ़तापूर्वक असहमत
1. A significant change has been improvement in confidence	1. उसके आत्मविश्वास में महत्त्वपूर्ण सुधार आया है।	☐	☐	☐	☐	☐
2. He was very dependent on us before implantation	2. इम्प्लांट से पहले वह हम पर बहुत अधिक निर्भर रहता था।	☐	☐	☐	☐	☐
3. I can seldom leave him to do something on his own	3. कभी-कभार ही इसको अपने से कुछ करने लिए छोड़ते है|	☐	☐	☐	☐	☐
4. He is as independent as most other children of his age	4. वह उतना ही आत्मनिर्भर है जितना कि उसकी उम्र के बच्चे आत्मनिर्भर होते है।	☐	☐	☐	☐	☐

WELL-BEING & HAPPINESS	हाल चाल तथा खुशी	दृढ़तापूर्वक सहमत	सहमत	ना सहमत ना ही असहमत	असहमत	दृढ़तापूर्वक असहमत
1. He continues to be a happy child and good fun to be with	1. वह पहले जैसा खुशमिजाज बच्चा है और उसके साथ रहना अच्छा लगता है।	☐	☐	☐	☐	☐
2. He is less frustrated than before he had the implant	2. इम्प्लांट से पहले की अपेक्षा में अब वह कम निराश रहता है।	☐	☐	☐	☐	☐
3. His behaviour has improved since he had his implant	3. जब इम्प्लांट हुआ है उसके व्यवहार और बरताव में काफी सुधार आया है।	☐	☐	☐	☐	☐
4. He still shows signs of frustration in his behaviour	4. उसके व्यवहार में वह अभी भी निराशा के लक्षण दिखते है।	☐	☐	☐	☐	☐
5. He has become argumentative since getting his implant	5. इम्प्लांट के बाद वह तार्किक हो गया है।	☐	☐	☐	☐	☐

EDUCATION	शिक्षा	दृढ़तापूर्वक सहमत	सहमत	ना सहमत ना ही असहमत	असहमत	दृढ़तापूर्वक असहमत
1. He is keeping up well with children of his own age at school	1. वह स्कूल में अपनी उम्र के बच्चों के साथ उन्हीं के समान हर काम अच्छे से करता है।	☐	☐	☐	☐	☐
2. He is unable to cope with mainstream schooling	2. वह मुख्य धारा के स्कूल में शिक्षा प्राप्त करने में असमर्थ है।	☐	☐	☐	☐	☐
3. He is totally reliant on his implant at school	3. वह स्कूल में पूर्णरूप से अपने इम्प्लांट पर निर्भर रहता है।	☐	☐	☐	☐	☐
4. The local school and support services adequately meet all our needs concerning the use of his implant at school	4. इम्प्लांट प्रयोग करने से सम्बंधित हमारी हर आवशकता को पूरा करने में स्थानीय स्कूल और सहायता सेवा केंद्र सक्षम रहा है।	☐	☐	☐	☐	☐
5. Parents should have a choice in the use of sign language at school	5. स्कूल में साकेतिक भाषा के चुनाव करने के लिए माता पिता के पास विकल्प और प्रावधान होना चाहिए।	☐	☐	☐	☐	☐
6. We feel the need for advice from the Implant Centre concerning his future	6. हमें लगता है कि इसके भविष्य के विषय में इम्प्लांट केंद्र से सलाह लेने की आवश्यकता है।	☐	☐	☐	☐	☐
7. We are reliant on the Implant Centre for technical advice about his implant	7. हम इसके इम्प्लांट के तकनीकी सलाह के लिए इम्प्लांट केंद्र पर निर्भर है।	☐	☐	☐	☐	☐
8. I am concerned about his future school placement	8. मैं इसके भविष्य के शैक्षिक व्यवस्था के बारे में चिंतित हूँ।	☐	☐	☐	☐	☐

EFFECTS OF IMPLANTATION	इम्प्लांट का प्रभाव	दृढ़तापूर्वक सहमत	सहमत	ना सहमत ना ही असहमत	असहमत	दृढ़तापूर्वक असहमत
1. I worry that the implant will break down	1. मुझे यह डर रहता है कि यह इम्प्लांट टूट या ख़राब हो जाएगा।	☐	☐	☐	☐	☐
2. The whole process of implantation is still stressful	2. इम्प्लांट कि पूरी प्रक्रिया अभी तक तनावपुर्ण रही है।	☐	☐	☐	☐	☐
3. I am happy about his progress at school	3. मैं स्कूल में इसके प्रगति से खुश हूँ ।	☐	☐	☐	☐	☐
4. Progress during the first few months seemed very slow	4. पहले कुछ महीनों के दौरान लग रहा था कि प्रगति काफी धीमे हुई।	☐	☐	☐	☐	☐
5. I am confident that long term electrical stimulation will not be a problem	5. मुझे विश्वास है कि इस बिजली के उपकरण को लंबे समय तक प्रयोग से कोई मुश्किल नहीं होगी।	☐	☐	☐	☐	☐
6. Immediately after implantation his ability to communicate was poorer	6. इम्प्लांट के तुरंत बाद उसकी संप्रेषण क्षमता काफी कमजोर थी।	☐	☐	☐	☐	☐

SUPPORTING THE CHILD	बालक की सहायता	दृढ़तापूर्वक सहमत	सहमत	ना सहमत ना ही असहमत	असहमत	दृढ़तापूर्वक असहमत
1. He has needed more help from me since he received his implant	1. जब से उसने अपना इम्प्लांट प्राप्त किया है, उसे मुझसे और मदद की आवश्यकता लगने लगी है।	☐	☐	☐	☐	☐
2. A parent of a child with an implant needs to be patient as benefits may take time to show	2. इम्प्लांट से लाभ दिखने में समय लग सकता है। बच्चे के माता-पिता को धैर्य रखने की आवश्यकता है	☐	☐	☐	☐	☐
3. I find it easier to communicate with him by speaking than by signing	3. मुझे उससे बोलकर संवाद करना, सांकेतिक भाषा से ज्यादा आसान लगता है।	☐	☐	☐	☐	☐
4. I give him the same amount of help as before his implant	4. मैं उसकी उतनी ही मदद करता हूँ जितनी की इम्प्लांट से पहले।	☐	☐	☐	☐	☐
5. The help I give him has become more productive now he has his implant	5. मैं उसकी जो मदद करता हूं अब उसके और भी अच्छे परिणाम है क्योंकि अब उसके पास उसका इम्प्लांट है।	☐	☐	☐	☐	☐
6. A lot of help at first means a child needs less help later	6. पहले बहुत मदद का मतलब है कि बच्चे को बाद में कम मदद की जरूरत होगी	☐	☐	☐	☐	☐
7. I get more time to myself because of his increased independence	7. उसकी बढ़ी हुई स्वतंत्रता के कारण, मैं अपने लिए अब अधिक समय निकाल पाता हूँ।	☐	☐	☐	☐	☐

 © 2025. The Author(s).

**DECISION OF IMPLANTATION**	**इम्प्लांट का निर्णय**	दृढ़तापूर्वक सहमत	सहमत	ना सहमत ना ही असहमत	असहमत	दृढ़तापूर्वक असहमत
1. Making the decision to proceed with implantation was the most difficult part	1. इम्प्लांट करने का निर्णय लेना, मेरे लिए पूरी परिक्रिया का सब से अधिक कठिन भाग था।	☐	☐	☐	☐	☐
2. I chose implantation for my child so he would have a chance to become part of the hearing world	2. मैंने अपने बच्चे के लिए इम्प्लांटेशन चुना ताकि उसे सुनने वालो की दुनिया का हिस्सा बनने का मौका मिले।	☐	☐	☐	☐	☐
3. I expected him to learn to talk once he had his implant	3. मुझे अपेक्षा है कि इम्प्लांट होने के बाद, वह बात करने लगेगा।	☐	☐	☐	☐	☐
4. Progress after implantation has exceeded my expectations	4. इम्प्लांटेशन के बाद बालक के व्यवहार में प्रगति मेरी अपेक्षाओं से अधिक हुई है ।	☐	☐	☐	☐	☐
5. I worry that ultimately, he may be neither part of the deaf nor the hearing world	5. मुझे चिंता है कि अंततः वह न तो बधिरों का हिस्सा होगा और न ही सुनने वालो की दुनिया का।	☐	☐	☐	☐	☐
6. It was a difficult time waiting for the results of the assessments before implantation	6. इम्प्लांटेशन से पहले की जाने वाली जांचो/परिक्षण के परिणामों की प्रतीक्षा करना एक बहुत ही मुश्किल समय था।	☐	☐	☐	☐	☐
7. It was a relief seeing him respond to sound for the first time	7. उसे पहली बार ध्वनि पर प्रतिक्रिया करते हुए देखना एक राहत की बात थी।	☐	☐	☐	☐	☐
8. I am concerned that my child will be rejected by the deaf community because of the implant	8. मुझे चिंता है कि इम्प्लांट के कारण मेरे बच्चे को बधिर समुदाय द्वारा खारिज कर दिया जाएगा।	☐	☐	☐	☐	☐
9. It was important to me that my child could hear sounds from traffic for safety	9. मेरे लिए यह महत्वपूर्ण था कि मेरा बच्चा सुरक्षा कारणों के लिए यातायात आवाजों को सुन सके।	☐	☐	☐	☐	☐
10. I believe now that my will have reasonable prospects for employment.	10. मुझे अब लगता है कि बच्चा पढ़ - लिख कर रोजगार प्राप्त कर सकेगा।	☐	☐	☐	☐	☐

**PROCESS OF IMPLANTATION**	**इम्प्लांट की प्रक्रिया**	दृढ़तापूर्वक सहमत	सहमत	ना सहमत ना ही असहमत	असहमत	दृढ़तापूर्वक असहमत
1. It has been a problem getting someone to look after the family when we go to the Implant Centre	1. यह एक बड़ी समस्या है कि जब हम इम्प्लांट केंद्र जाते है तो घर पर परिवार की देखभाल के लिए कोई नहीं होता है।	☐	☐	☐	☐	☐
2. The process of implantation was no more intrusive than expected	2. इम्प्लांट की प्रक्रिया जितनी मुश्किल लग रही थी उतना ज्यादा मुश्किल नहीं थी।	☐	☐	☐	☐	☐
3. Travelling to implant Centre is a burden on our time	3. इम्प्लांट केंद्र पर जाने के लिए समय निकालना मुश्किल होता है।	☐	☐	☐	☐	☐
4. The costs of travel to the Implant Centre are a problem	4. इम्प्लांट केंद्र पर आने जाने का खर्चा एक समस्या है।	☐	☐	☐	☐	☐
5. It is important to encourage children to listen through their implants at home	5. यह जरूरी है कि बच्चों को घर पर इम्प्लांट के द्वारा सुनने के लिए प्रोत्साहित करें।	☐	☐	☐	☐	☐
		☐	☐	☐	☐	☐
6. A positive attitude is a great help towards successful use of the implant	6. सकारात्मक रवैया, इम्प्लांट के सफल प्रयोग के लिए बहुत मदद करता है।	☐	☐	☐	☐	☐
7. Regular tuning and checking of the implant system are essential	7. नियमित रूप से इम्प्लांट की ट्यूनिंग तथा जाँच अत्यंत आवश्यक है।	☐	☐	☐	☐	☐
8. Only experienced teams should carry out cochlear implantation	8. केवल अनुभवी टीमों को कोक्लियर इम्प्लांट करना चाहिए।	☐	☐	☐	☐	☐
9. The most important factor in choosing an implant device is its reliability	9. इम्प्लांट डिवाइस चुनने में सबसे महत्वपूर्ण कारक इम्प्लांट की विश्वसनीयता है।	☐	☐	☐	☐	☐
10. At least one visit per year by Implant Centre staff to home/school is essential	10. कम से कम साल में एक बार इम्प्लांट केंद्र से कोई कर्मचारी, स्कूल या घर पर निरीक्षण के लिए आना आवश्यक है।	☐	☐	☐	☐	☐
11. I wish to participate in meetings with other families having an implanted child	11. मैं ऐसे परिवारों के बैठक में भाग लेना चाहूंगा। जिनके बच्चों को इम्प्लांट हुआ है।	☐	☐	☐	☐	☐
12. Other children in the family resented the time and attention taken up by the implant	12. परिवार के अन्य बच्चों ने प्रत्यारोपण द्वारा लिए गए समय और ध्यान पर नाराजगी जताई।	☐	☐	☐	☐	☐
13. Before proceeding with implantation, parents should obtain as much information and advice as possible	13. इम्प्लांट करवाने से पहले माता पिता को इम्प्लांट से संबंधित अधिक से अधिक जानकारी/सलाह लेनी चाहिए।	☐	☐	☐	☐	☐
14. The programme at the Implant Centre should emphasize speaking and listening	14. इम्प्लांट केंद्र में होने वाले कार्यक्रमों में सुनने तथा बोलने पर अधिक से अधिक ज़ोर देना चाहिए।	☐	☐	☐	☐	☐
15. Signing support is helpful for a considerable time after implantation	15. इम्प्लांट के बाद एक उचित समय तक सहायक सांकेतिक भाषा काफी उपयोगी होते है।	☐	☐	☐	☐	☐
16. It was useful to meet another family with an implanted child before deciding on an implant	16. इम्प्लांट कराने का फैसला लेने से पहले उन परिवारों से मिलना जिनके परिवार में इम्प्लांट हो चुका है। काफी उपयोगी होता है।	☐	☐	☐	☐	☐

| इस प्रश्नावली को पूरा करने के लिए, आपका बहुत बहुत धन्यवाद |

 © 2025. The Author(s).
